# Glial Cells—The Strategic Targets in Amyotrophic Lateral Sclerosis Treatment

**DOI:** 10.3390/jcm9010261

**Published:** 2020-01-18

**Authors:** Tereza Filipi, Zuzana Hermanova, Jana Tureckova, Ondrej Vanatko, Miroslava Anderova

**Affiliations:** 1 Department of Cellular Neurophysiology, Institute of Experimental Medicine, Academy of Sciences of the Czech Republic, 14200 Prague, Czech Republic; tereza.filipi@iem.cas.cz (T.F.); zuzana.hermanova@iem.cas.cz (Z.H.); jana.tureckova@iem.cas.cz (J.T.); ondrej.vanatko@iem.cas.cz (O.V.); 2 2nd Faculty of Medicine, Charles University, 15006 Prague, Czech Republic

**Keywords:** ALS, astrocytes, microglia, oligodendrocytes, NG2-glia, pericytes, clinical trials

## Abstract

Amyotrophic lateral sclerosis (ALS) is a fatal neurological disease, which is characterized by the degeneration of motor neurons in the motor cortex and the spinal cord and subsequently by muscle atrophy. To date, numerous gene mutations have been linked to both sporadic and familial ALS, but the effort of many experimental groups to develop a suitable therapy has not, as of yet, proven successful. The original focus was on the degenerating motor neurons, when researchers tried to understand the pathological mechanisms that cause their slow death. However, it was soon discovered that ALS is a complicated and diverse pathology, where not only neurons, but also other cell types, play a crucial role via the so-called non-cell autonomous effect, which strongly deteriorates neuronal conditions. Subsequently, variable glia-based in vitro and in vivo models of ALS were established and used for brand-new experimental and clinical approaches. Such a shift towards glia soon bore its fruit in the form of several clinical studies, which more or less successfully tried to ward the unfavourable prognosis of ALS progression off. In this review, we aimed to summarize current knowledge regarding the involvement of each glial cell type in the progression of ALS, currently available treatments, and to provide an overview of diverse clinical trials covering pharmacological approaches, gene, and cell therapies.

## 1. Introduction

Amyotrophic lateral sclerosis (ALS) is a motor neuron disease (MND), resulting in the progressive degeneration of the upper and lower motor neurons (MNs) in the motor cortex, the brain stem, and the spinal cord. The paralysis begins focally, depending on the location of primary pathology [1], and it disseminates in a pattern suggesting that the degeneration is spreading among adjacent pools of MNs [2]. Survival is highly variable, as ALS manifests in different ways and it has been recognized for many years that the different clinical presentations correspond with differences in survival [3]. However, the respiratory failure usually leads to death about 3–4 years after onset [4,5]. In spite of the poor prognosis, there are also studies showing better survival rates [6,7], although there is still no effective cure that is available to stop or delay the disease from progression. The only therapeutic agents approved are Riluzole, and, also in some countries, Edaravone (also termed Radicava, previously MCI-186). They both only slightly prolong the survival of ALS diagnosed patients.

Traditionally, ALS is classified as either the sporadic (sALS) or familial form (fALS), while the majority of cases among the population are sporadic. The term sporadic refers to ALS that occurs without a family history of ALS, but it can sometimes be mistakenly thought to refer to ALS occurring without a genetic basis. On the contrary, it is now evident that 1–3% of sALS cases reflect mutation in cytosolic superoxide dismutase 1 (SOD1) [8] and another 5%, or even more, are caused by intronic expansions in chromosome 9 open reading frame 72 (C9ORF72) [9]. Mutations in other ALS related genes (e.g., *FUS*, *TARDBP*, *OPTN*) have also been identified in the sALS cases, although they are rare. The first ALS-related gene, *SOD1*, was reported in 1993 [10], and, since then, more than 30 genes were found to cause ALS in the case of their mutation. These findings led to the establishment of several animal models of the disease, which can improve our understanding of the mechanism of ALS pathophysiology and possibly lead to the generation of new therapeutic approaches.

In this study, we discuss certain animal models, individual glial cells, and their participation in the pathology. We also present an overview of clinical trials that have been made in previous years, in addition to those that are currently running or recruiting participants.

## 2. Clinical Phenotype 

A characteristic motor deficit of ALS can affect any voluntary muscle, which results in a heterogeneous presentation of symptoms. The symptoms usually have focal onset, but the disease eventually spreads to other regions. According to Gowers’ clinical observation [11], the spreading seems to be both local (within the same limb) and also between neuro-anatomically linked regions (rostral-caudal or contra-lateral). The heterogeneous clinical manifestations and variable speed of progression make the diagnosis difficult. Studies have shown that at least 10% of ALS patients were misdiagnosed, despite being rather common MND [12], especially in the early stages, due to nonspecific symptoms that resemble other neurological diseases. The correct identification of specific ALS phenotype has important implications for patients, particularly with regards to prognosis and survival, and also for their possible enrolment in clinical trials.

Amyotrophic lateral sclerosis comprises two main clinical manifestations. *Limb-onset ALS* is characterized by a combination of upper and lower MN degeneration signs in limbs, and *bulbar-onset ALS* is characterized by dysarthria, dysphagia (which can develop later or simultaneously with dysarthria), and also with limb features developing later. Less common are *primary lateral sclerosis*, which mostly affects corticospinal MNs and is accompanied with symptoms, like hyperreflexia, spasticity and increased limb tone, but with relatively little muscle atrophy [13,14] and *progressive muscular atrophy*, which mainly affects the lower MNs, and patients suffer from weakness, flaccidity, and atrophy of the limbs [15,16]. ALS also shows clinical overlap with some other adult-onset disorders, usually frontotemporal dementia (FTD). Atypical presentation of clinical symptoms may further include weight loss, rapid emotional changes that are associated with frontal lobe-type cognitive dysfunction, and muscle cramps and fasciculation without muscle weakness [17]. The present clinical diagnosis mainly relies on the identification of related upper and lower MN dysfunction with clear disease progression across different body regions, despite the enormous effort to discover reliable diagnostic markers identifying ALS in its early stages. This current approach is time consuming, resulting in a definitive diagnosis between eight and fifteen months [18]. Due to the complexity of ALS symptoms and their variability within the population of ALS patients, misdiagnosing might also occur as in the case of demyelinating motor neuropathies. Though, recent findings that added new tiles into mosaic of ALS pathology may point towards other possible therapeutic targets; for example, miR-218 in MNs [19], astrocytic metabolic profile and the membrane transport of mitochondrial specific energy substrates [20], or astrocytic SERCA pump and store-operated Ca^2+^ entry [21]. In addition, recent progress in human induced pluripotent stem cell-based models enables studying the genetic factors that are related to ALS in patient-derived motor neurons and glial cells [22].

## 3. Pathophysiology of ALS

Resolving the relationship between upper and lower motor neuron dysfunction appears to be crucial in understanding ALS pathogenesis. Three distinct theories of ALS onset have been proposed [23]. *The dying forward hypothesis* suggested that ALS originates at a cortical level, with hyperexcitability of cortical MNs, which mediates neuronal degeneration via a trans-synaptic anterograde mechanism [24]. A contrasting theory, *the dying back hypothesis*, suggested dysfunction of lower MNs as a primary event, which begins within muscles or at the neuromuscular junction, and toxic factors are transported from the periphery to the axon cell body [25]. The third one proposed an independent and random degeneration of upper and lower MN [26,27]. The development of new techniques, such as threshold tracking transcranial magnetic stimulation, approved that cortical hyperexcitability represents an early pathophysiological feature of ALS, which potentially mediates MN degeneration via a dying-forward, trans-synaptic, glutamatergic mechanism [28]. 

The MN degeneration, followed by their death in the anterior horns of the spinal cord, brainstem motor nuclei, and motor cortex, represents a common denominator within the complexity of ALS. The degeneration of spinal MNs leads to muscle atrophy and the results in scarring in the lateral tracts of the spinal cord. Interestingly, in the early phases of the disease ALS spares the MNs, which innervate extraocular muscles, as well as those that control bowel and bladder function. During the progression, the affected spinal MNs shrink and accumulate rounded or thread-like deposits of aggregated proteins that are collectively referred to as inclusions. The inclusions in cytoplasm are often ubiquitinated. The transactive response DNA binding protein (TDP43) is an early target for ubiquitination, which is the major component of ubiquitinated inclusions in most ALS cases [29].

In ALS, the MN degeneration goes together with signs of oxidative stress and mitochondrial dysfunction, inclusion bodies, impairment of RNA processing, neurofilament aggregation, loss of axonal transport, disruption of the neuromuscular junction, and axon demyelination [30]. 

The development of ALS seems to be caused by numerous interactions of molecular and genetic pathways. Impaired glutamate uptake from the synaptic cleft leads to glutamate excitotoxicity due to the dysfunction of the glial excitatory amino acid transporters, which triggers neurodegeneration through the activation of Ca^2+^-dependent enzymatic pathways. In addition, mutations in the *SOD1*, *C9ORF72*, *TARDBP*, and *FUS* genes result in dysregulated RNA metabolism, which leads to abnormalities of translation and formation of intracellular neuronal aggregates. Mutations in the *SOD1* gene also increase oxidative stress and induce mitochondrial dysfunction and defective axonal transportation. For example, ALS cases that are caused by microsatellite expansions in *C9ORF72* show intranuclear RNA foci [31], distinctive cytoplasmic inclusions that are derived from dipeptide repeat proteins (DPRs) [32,33], as well as p62-positive; largely TDP-43-negative neuronal cytoplasmic inclusions that predominantly occur in the cerebellum and hippocampus [34]. Cases of ALS that are caused by mutations in *SOD1* and *FUS* are pathologically different. They do not exhibit TDP-43 pathology, but rather inclusions of abnormal SOD1 and FUS proteins. In addition to the pathological findings in MNs, there is abundant evidence of a significant pathology in non-neural cell types, such as the appearance of reactive astrocytes and activated microglia, which secrete neurotoxic factors and pro-inflammatory cytokines [35]. As reviewed below, it is likely that both forms of non-cell autonomous cellular reactivity adversely influence the ALS progression. Numerous model systems were developed, including in vitro biochemical systems, cell cultures, invertebrates, non-mammalian vertebrates, rodent models, and also recently, human patient-derived stem cell models, to study the pathological mechanisms of ALS.

## 4. ALS Models

Here, we briefly summarize the basic groups of ALS models and the advantages and disadvantages of their use, to provide the basic overview, since we mention the majority of these models in the chapters describing the role of individual glial cells in ALS. We recommend excellent reviews by [36,37,38,39] and guidelines for preclinical animal research in ALS by [40] for readers who are more interested in all ALS models and their use in various studies. 

Genetic models are based on the known mutations of ALS causative genes. Table 1 and Table 2 provide a list of the most frequently used ALS genetic models, along with the relevant gene mutations and the affected functions. 

SOD1 transgenic models. The mutation of the gene for SOD1 was the first to be identified as a cause of ALS [10,41] and Gurney et al. generated the first SOD1 mouse model in 1994 [42]. Since then, more than ten models that are based on the overexpression of the mutated form of this gene have been generated [39]. All of the mutant SOD1 (mSOD1) mouse models develop progressive MN degeneration, similar to the human disease, varying in the onset of symptoms as well as progression rates. Moreover, astrogliosis, microgliosis, impaired glutamate uptake, mitochondrial vacuolization, and decreased metabolic support from glia to MNs occur in these models [38]. The absence of degeneration of MNs in the cerebral cortex, which is one of the main features of human disease and also one of the diagnostic aspects, is a major disadvantage of models with SOD1 mutation. Moreover, these mice have a tendency to spontaneously reduce the number of transgene copies, which can affect the phenotype of this model [37,43]. Corresponding rat models have also been generated [44,45,46]. In addition, rodent models in which mSOD1 is selectively expressed in particular types of glial cells or neurons have been generated, to improve the understanding of the contribution of these cell types to ALS pathology [47,48,49,50,51,52], for review see [53]. Aside from rodents, various SOD1 zebrafish models were also developed [54,55]—see Table 1.

TDP-43 and FUS transgenic models. A common feature of TDP-43 and FUS models is that they are based on mutations in the genes (TARDBP, FUS) for RNA-binding proteins (TDP-43, FUS/TLS), both of which are involved in RNA processing and modifications. Similarly to the SOD1 models, these models exhibit progressive motor dysfunction, MN degeneration, impaired neuromuscular integrity, astrocytosis, and microglia infiltration. In addition, they are characterized by the presence of cytoplasmic inclusions positive for TDP-43 and ubiquitin (for review see [56]). A variety of TDP-43 models have been generated, which differ in the type of mutation, the used promoter, as well as the level of overexpression—see Table 2. Similarly, rat models overexpressing human mutant TDP-43 M337V were developed [57]. Interestingly, models performing neuron specific mutations of TARDBP were not shown to develop progressive MN degeneration. On the other hand, using the astrocyte specific TDP-43 mutation model (M337V) [58] suggested a non-cell autonomous mechanism of neuronal death. The main limitations of using these models lie in their dependence on the level of overexpression. Only mild phenotypes are observed in the low overexpression models, and the early mortality of such animals is not due to progressive muscle atrophy [59]. On the other hand, in high overexpression models, the symptoms occur, even in the wild type TDP-43 mutants. Zebrafish TDP-43 models (A315T or Q331K) show defects in MN function, axonopathy, and impaired branching [39,60].

C9ORF72 models. An excessive hexanucleotide (GGGGCC) repeat expansion in the first intron of C9ORF72 accounts for approximately 40% of fALS [31]. Several recent studies have described different approaches for developing the C9ORF72 models, but neither the C9ORF72 knockout mice [61] nor different knockouts [62,63] were successful. Another approach—bacterial artificial chromosomes (BAC) transgenic mice with human C9orf72 expansion repeat—was described by four groups [64,65,66,67], for review see [68]. 

Induced pluripotent stem cells (iPSCs) that were derived from ALS patients comprise of a special group of ALS models [69]. These models have many advantages when compared to animal ALS models. The main advantage is the natural endogenous mutation, eliminating the need for the overexpression of genes carrying the pathogenic mutation. This model is one of the few that allows for the study of the sALS. Furthermore, iPSCs can be differentiated into specialized cell types, such as MNs [70].

Wobbler mice represent an independent group of ALS models, in which the mutation causing symptoms of the disease is spontaneous. The wobbler mouse spontaneously arose in a C57BL/Fa strain and the autosomal recessive-mutation is caused by a point mutation in the Vps54 gene. Although the wobbler mouse is the best-characterized spontaneous mutant with a progressive degeneration of upper and lower motor neurons and showing striking similarities to ALS patients, the comparable mutation of VPS54 has not been found in ALS patients yet, for review see [71].

## 5. The Role of Glia

The genes, of which mutations cause ALS, are expressed in multiple cell types. This means that the disease arises from a damage combination within MNs and their glial partners, rather than exclusively from neuronal damage. For mSOD1, this statement is supported by studies using a mouse model of ALS, which revealed that ALS does not develop when its expression is restricted to neurons [48,49]. In addition, the ALS progression was slowed down when the mSOD1 was conditionally deleted in individual glial populations (microglia, astrocytes, or oligodendrocytes) [99,100,101]. On the other hand, when the level of mSOD1 in MNs was reduced, no beneficial effect was shown in slowing the rate of disease progression after onset, not even when its level was reduced pre-symptomatically [99,101,102]. Such findings point towards the fact that glial cells might represent the key determinants of disease symptoms onset and/or progression. While most of the early findings on glial involvement in ALS pathogenesis were derived from studies on mutant SOD1, there is also accumulating evidence for glial contribution in other subtypes of ALS [103]. The development of reactive gliosis—the accumulation of the morphologically and functionally altered glial cells that are involved in the neuro-immune response—is a key feature of neuroinflammation. During an acute neuro-immune response, microglia can adopt an “M1” pro-inflammatory phenotype or “M2” protective/anti-inflammatory function, depending on the type of insult [104]. Similarly, reactive astrocytes can both contribute to (“A1”) and prevent (“A2”) damage from inflammation [105,106].

## 6. Astrocytes

Astrocytes are the largest population of non-neuronal cells in the central nervous system (CNS) and they possess a wide range of functions in a healthy brain. They provide metabolic support for neurons, ionic-, and neurotransmitter homeostasis and they are involved in the maintenance of blood brain barrier (BBB) integrity. Therefore there is increasing evidence that astrocytes strongly contribute to neurodegeneration, and our understanding of the processes, which occur in the damaged CNS, is crucial for potential therapy development.

### 6.1. Downregulation of Glutamate Transporters in Astrocytes

Glutamate buffering is one of the astrocytic physiological features. Clearing glutamate from excitatory synapses is essential in normal synaptic transmission and its impairment leads to the damage of neurons. In healthy tissue multiple excitatory amino acid transporters (EAATs) mediate the glutamate transport. Those mainly expressed on astrocytic membranes are EAAT1 and EAAT2. In normal tissue, they take up the majority of synaptic glutamate and their transporting ability is driven by the electrochemical gradient of Na^+^ (for a review see [107]). During ALS progression, astrocytes may lose the majority of EAAT2 (in murine models referred to as GLT-1) in the spinal cord of mSOD1^G93A^ murine models, as well as in animals with transplanted human neural progenitors [45,108,109]. Interestingly, during development, the level of EAAT2 in glial-restricted progenitors (which differentiate into astrocytes) is higher in murine mSOD1^G93A^ models, when compared to the controls; moreover, these cells do not yet show any signs of reactive astrogliosis [108,110].

**Figure 1:**
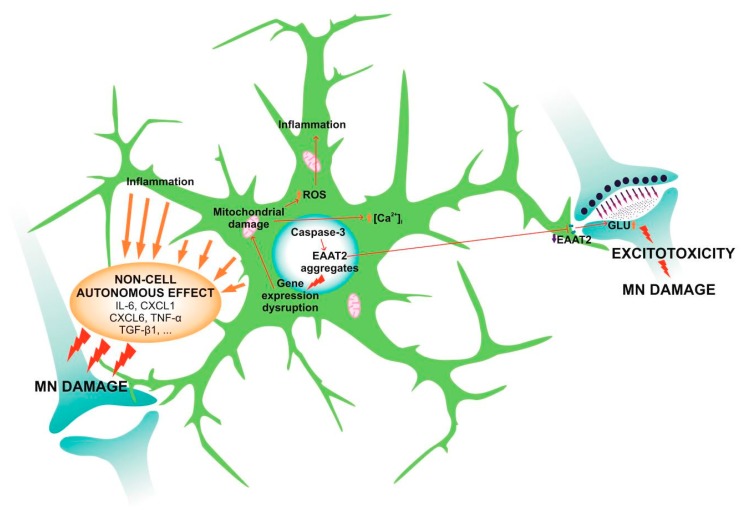
During ALS astrocytes undergo pathological changes, which affect their physiological functions and lead to the reduction of motor neuron (MN) survival. ALS astrocytes have decreased levels of EAAT2, which are cleaved by Caspase-3 and create aggregates within astrocytic nucleus. Therefore, the number of EAAT2 receptors on membranes is reduced and the astrocytic ability to buffer glutamate (GLU) from synapses is impaired. Increased levels of GLU cause excessive neuronal stimulation that damages MNs via the process termed excitotoxicity. The nuclear aggregates not only damage MNs but also astrocytes themselves, as they cause gene expression disruption and subsequently, mitochondrial damage. Moreover, astrocytes secrete a range of inflammatory soluble factors in response to the intracellular damage (interleukins, cytokines...), which strongly reduce MN survival via the so-called non-cell autonomous effect.

Caspase-3, which generates two fragments from EAAT2 protein, mediates the glutamate transport [111]. These are then sumoylated and accumulate within the astrocytic nuclei in the spinal cord. The accumulation of EAAT2 fragments coincides with the disease progression [112]. This process causes morphological changes within the astrocytes and the EAAT2 protein aggregates in cellular nuclei dysregulate astrocytic gene expression. The most affected genes are related to mitochondrial functions and cellular respiration [113]. Damaged mitochondria lack the ability to buffer intracellular Ca^2+^ and its cytosolic concentration increases, together with the concentration of reactive oxygen species (ROS) ([114]; Figure 1).

The decrease in the level of EAAT2 leads to the deterioration of glutamate buffering and its transport into the astrocytes. Therefore, glutamate accumulates in the synaptic cleft and it causes excessive and pathological neuronal stimulation, which disrupts ionic homeostasis in the neurons. This process is termed glutamate excitotoxicity and contributes to MNs damage and death in ALS [112,113,115]. In both mice and humans, the loss of EAAT2 proteins does not appear until the symptomatic stage of the disease [116], which means that it is slightly delayed relative to the changes in astrocytes towards reactive phenotype [113].

### 6.2. Reactive Astrogliosis

In neurodegenerative diseases, such as ALS, astrocytes change their morphology and function and then become reactive in response to various stimuli, for example soluble factors that are secreted by microglia [106]. This process comprises of extensive molecular changes that lead to astrocyte hypertrophy and proliferation and to secretion of pro- and anti-inflammatory cytokines, chemokines, interferons, and growth factors, together with components of extracellular matrix [117]. Such activation has a dual effect. It limits the spread of the lesion and restricts ongoing inflammation by preventing infiltration of activated immune cells into the surrounding tissue and, thus, reduces subsequent neuronal degeneration [118]. On the contrary, the modifications of extracellular matrix, which are an essential part of reactive astrogliosis and glial scar formation, contribute to the inhibition of axonal regeneration and growth [117].

However, the activated astrocytes in ALS have slightly different properties, which are common for astrocytes from human sALS and fALS and murine models [109,119]. Astrocytes that are isolated from mSOD1^G93A^ murine models have higher proliferative potential in vitro than the wild-type astrocytes [120] and the first reactive cells appear before the disease symptoms manifestation and MN degeneration [45]. The mSOD1^G93A^ astrocytes are larger than those in healthy tissue, with more hypertrophied processes [109]. They express markers that are typical for astrogliosis as well as markers of immature astrocytes, such as high levels of non-filamentous glial fibrillary acidic protein (GFAP) [108,109]. In addition, the expression of connexin 43 (Cx43), which is generally elevated in reactive astrocytes [121], shows a similar increase in mSOD1^G93A^ astrocytes. During the pre-symptomatic stages of the ALS disease, Cx43 in astrocytes is only slightly increased above the physiological level. This elevation becomes more substantial during disease progression [116,120,122]. In addition to the above-mentioned proteins and changes, mSOD1^G93A^ astrocytes overexpress the α2 subunit of Na^+^/K^+^ ATPase [123], which directly interacts with astrocytic glutamate transporters and affects their functioning via an electrochemical gradient [124,125]. How this change in Na^+^/K^+^ ATPase expression is affected by the reduction of EAAT2 levels (and vice versa) remains to be elucidated. However, Gallardo and colleagues proposed that the level of α2-Na^+^/K^+^ ATPase is elevated in response to mitochondrial damage, and it is supposed to anticipate reduced ATP levels within the astrocytes by the stimulation of mitochondrial respiration. Subsequent to an increase in cellular respiration, ROS levels increase within the astrocytes and activate an inflammatory response, causing non-cell autonomous MN degeneration [123].

### 6.3. Non-Cell Autonomous Effect

Activated astrocytes lack the ability to support the survival and recovery of MNs [106,126]. In fact, they do the exact opposite. They increase MN damage by the so-called non-cell autonomous effect, which has been confirmed in several studies using astrocyte-conditioned media for MN cultivation. Results from both in vitro and in vivo studies with murine (SOD1^G93A,G37R,G86R^, C9ORF72, and TDP-43^A315T^ mutants) and human tissue showed significantly declined neuronal survival [109,127,128,129,130,131,132]. This process is mediated by astrocyte-specific soluble factors. Several molecules were identified that affect the physiological functioning of MNs in ALS, such as cytokines or growth factors (IL-6, CXCL1, 10 and 12, tumor necrosis factor-alpha (TNF-α) or transforming growth factor-beta (TGF-β1)). These molecules are upregulated in ALS astrocytes (together with prostaglandin D2 receptor, Sonic hedgehog (SHH), SHH-responsive genes, etc.) and they are secreted to the surrounding tissue [74,120,131,133,134,135], as in Figure 1. They cause alterations in MN morphology, namely smaller cellular bodies and shorter axons [74,131]. Apart from morphological changes, these molecules cause axonal swelling and the accumulation of mSOD1 and ubiquitin-positive aggregates in axons and somata of MNs [74,131]. The aggregates appear even before the onset of disease symptoms and their level continues to rise during ALS development [116], which corresponds with the progress of reactive astrogliosis. The mSOD1 protein is suspected of contributing to MN degeneration via the impairment of mitochondrial functions (hereby the intracellular levels of ROS and Ca^2+^ are increased and trigger an MN inflammatory response—for a review see [136]), together with increasing nitrosative stress [127,128,129,132,137]. Damaged mitochondria then become permeable, which leads to the release of ‘pro-cell death factors’, and subsequently to MN necroptosis [138]. Aside from MNs, astrocytic soluble factors also affect the functioning of other cell types, such as microglia, and regulate their immunological responses [139].

### 6.4. Astrocytes in C9ORF72 and TDP-43 Pathology

Similarly to the mSOD1 astrocytes, human and murine-derived astrocytes with C9ORF72 or TDP-43 pathology also show changed physiological properties and affect functioning of surrounding cells, especially MNs. The expansion of a non-coding GGGGCC repeats in C9ORF72 in astrocytes strongly affects RNA metabolism. The transcription of the hexanucleotide repeat leads to the formation of aggregates of poly-proline-arginine peptides, which bind other mRNAs within astrocytic nucleus. These aggregates then further affect- and often block-RNA splicing and nuclear export and, subsequently, protein transcription as well [140,141,142].

These changes in protein translation lead to a reduction of the metabolic flexibility of C9ORF72 astrocytes, together with an inhibition of proteasome functions and autophagy pathways and eventually to the activation of heat shock protein (HSP)-response [20,143,144]. Allen and colleagues also found a reduced ability of the astrocytes to metabolize glycogen and thus to utilize cellular energy sources [145]. Another change that is common for both mSOD1 and C9ORF72 astrocytes is the reduced expression of glutamate transporters, which causes a reduction in glutamate buffering, leads to glutamate accumulation in synaptic clefts, and causes excitotoxicity to MNs [146]. Similar changes in glutamate signalling were described also in C9ORF72 Drosophila model of ALS [147]. C9ORF72 astrocytes contribute to the MN degeneration in the ALS also via the release of soluble factors (non-cell autonomous effect), as mentioned above [148,149]. Aside from single soluble molecules, C9ORF72 astrocytes release extracellular vesicles, containing specific microRNAs, which (after contact with several specific targets, such as semaphorin proteins) cause axonal retraction and worsen overall MN survival [150].

On the contrary, the information regarding the role of astrocytes with mutated TDP-43 in ALS is unclear. It was showed that, within astrocytes, intracellular cytoplasmic inclusions, called stress granules, are formed. Their formation increases with the time and eventually leads to the cell death [151,152,153,154]. These granules consist of insoluble phosphorylated TDP-43 protein together with ubiquitin and α-synuclein [151,155,156]. Interestingly, the changes in TDP-43 expression and its solubility were also observed in mSOD1^G93A^ model of ALS, where TDP-43 created similar cytoplasmic aggregates [157], as well as in the glial cells of Drosophila TDP-43^D169G, G298S, A315T, N345K^ model of ALS [158]. Similarly to the other ALS models, the TDP-43^M337V^ astrocytes show increased oxidative damage thanks to the impairment of glutathione antioxidant function [159] and their phenotype changes towards the reactive one [160,161], which correlates with the global inflammation and increase in the levels of components of the innate immune complement system in TDP-43^Q331K, A315T^ pathology [162,163]. The reactive astrocytes then respond with an upregulation of small HSPs [164], which usually serves as a protective and stress response to cellular damage [165]. In addition, the number of astrocytes in the motor cortex of TDP-43^A315T^ murine models is higher than in healthy tissue [163].

TDP-43 cells were shown to contribute to MNs degeneration via the already-discussed non-cell autonomous effect, which is in agreement with the results from different ALS murine models [58,134], as well as Drosophila model [166]. However, the role of TDP-43 astrocytes in MN degeneration is not so clear. Several groups obtained different results. Their results demonstrated that when co-cultured with MNs TDP-43^M337V, A315T^ astrocytes do not show a non-cell autonomous effect and are not toxic to MNs [152,153].

Taken together, astrocytes play an important role in the pathology of ALS in human as well as in murine tissue. They affect numerous cell types, but mainly MNs, whose survival is strongly reduced due to astrocytic influence. As the role of astrocytes in ALS is not fully understood, these cells represent an important target for further research towards possible therapy development.

## 7. Microglia

As the immune-competent cells of the brain and spinal cord, microglia play an important role in maintaining normal CNS function. They colonize the brain early in development and transform into a highly ramified phenotype, constantly screening their environment. Microglia are activated by any kind of pathological event or change in the homeostasis of the CNS. The activation process is diverse and it depends on the context and type of the stressor/pathology. They influence the pathological outcome or response, owing to the release of plenty of substances, e.g., cytokines, chemokines, or growth factors.

**Figure 2:**
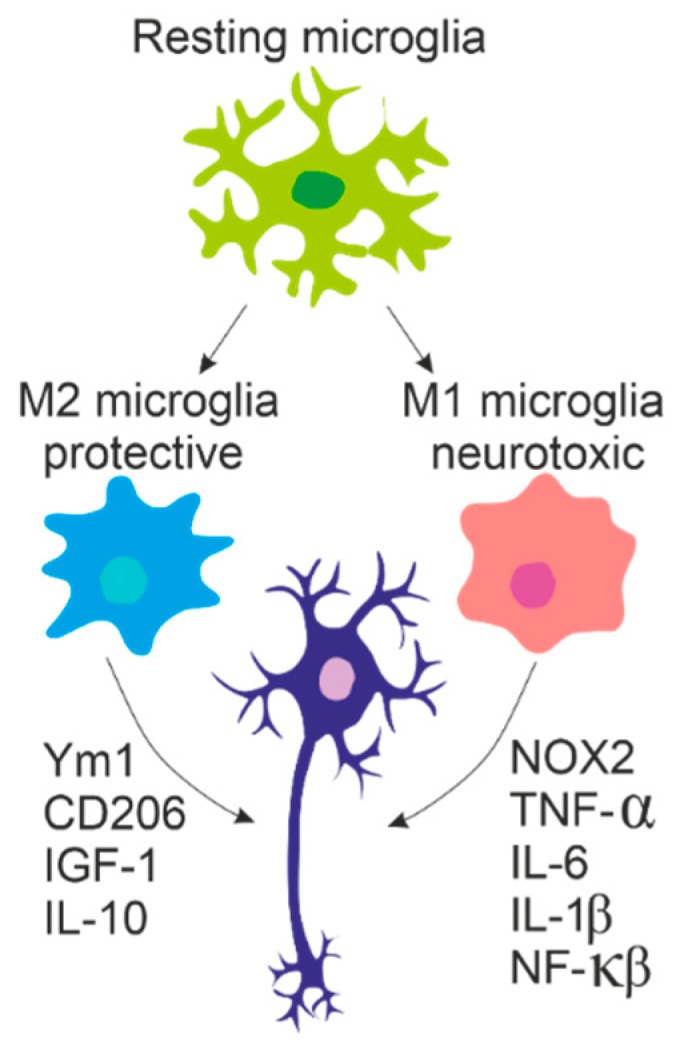
M2 and M1 microglia release certain molecules that are able to influence the survival of MNs. The molecules and their effect depend on the phenotype of microglia. M1 are toxic, while M2 microglia are protective.

Microglia become activated in all instances of ALS and there are numerous studies that have confirmed the presence of microglial activation at the site of MN damage in both ALS patients [167,168] and mSOD1 transgenic mice. Furthermore, increased microglial activation in the motor cortex has been shown to correlate with the severity of upper MN degeneration signs [168]. Studies using animal models indicate that in vivo resident microglia increase their number with the disease progression and their activation states represent a continuum between the two classical microglial phenotypes—neuroprotective M2 and toxic M1 [169,170], see Figure 2. In agreement with the occurrence of two different phenotypes on the basis of their morphology, Ohgomori and his team [171] described different types of microglia in mSOD1^G93A^ during ALS progression. The type “R1” was found in the early stage and it was poorly branched with short processes. “R2” type was similar to “R1” and only transiently occurred in the middle stage of the disease. The microglia appearing in the end stage, called “R3”, exhibited short and thick processes and large cell bodies [171].

### 7.1. Microglia in SOD1 Pathology

Microglia exhibit an anti-inflammatory profile, an overexpression of IL-10 and, moreover, attenuated Toll-like receptor 2 (TLR2) responses to a controlled immune challenge, in the pre-symptomatic stage of SOD1-mediated ALS [172]. At the disease onset, the expression of M2 markers Ym1 and CD206 was upregulated in the lumbar spinal cords of ALS mice, which indicated that microglia at this stage display an M2 phenotype. Eventually, in the final phase, microglia expressing high levels of NOX2, seem to prevail. NOX2 is the subunit of NADPH oxidase expressed by macrophages and is considered an M1 prototypic marker [173]. As the disease progresses, mSOD1^G93A^ expressing microglia undergo phenotypic transformation. More specifically, when co-cultured with MNs early stage M2 microglia enhance MN survival, whereas end-stage M1 microglia show toxic properties, which increases neuronal death [170]. In addition, mSOD1 microglia exhibit increased expression of molecular players of the endoplasmic reticulum (ER) stress pathway [174], such as C/EBP homologous protein (CHOP), which might be involved in their toxic phenotype.

One of the hallmarks of ALS is neuroinflammation, and M1 microglia seem to be hyper-reactive to inflammatory stimuli [175]. Nuclear factor-kappa β (NF-κβ) protein, which plays a key role in regulation of the inflammation, is upregulated in the spinal cords of ALS patients and mSOD1^G93A^ mice. It was shown that selective NF-κβ inhibition in ALS astrocytes is not sufficient for rescuing MNs from their death. However, the localization of NF-κβ activity and subsequent deletion of NF-κβ signaling in microglia rescued MNs from microglial-mediated death in vitro and extended the survival of ALS mice by impairing pro-inflammatory microglial activation. On the contrary, the constitutive activation of NF-κβ, selectively in wild-type microglia induced gliosis and death of MNs in vitro and in vivo [176]. The stimulation of excessive extracellular production of superoxide is another mechanism of mSOD1 damage that is produced by microglia. The SOD1 enzyme is not just catabolic, but it can also regulate NADPH oxidase–dependent superoxide production due to binding Rac1. This protein is a small GTPase controlling the NADPH oxidase activation. Inhibition of GTPase activity due to mSOD1^G93A^ results in the production of high levels of extracellular superoxide [177].

### 7.2. Microglia in C9ORF72 Pathology

The microglial function in ALS, which is associated with the mutations in *C9ORF72*, also undergoes some disturbances. Once it was known that the *C9ORF72* mutation results in decreased expression levels of this protein in ALS patients [31], it led to speculation that the loss of the C9ORF72 protein function might contribute to the disease onset/progression. The protein that is encoded by *C9ORF72* is probably a guanine exchange factor for one or more not-yet-identified G proteins. When inactivated in mice, abnormal microglia and age-related neuroinflammation occurs, which provides evidence that non-cell-autonomous, microglia-mediated inflammation might contribute to ALS [62,64,178]. Microglia have a proinflammatory phenotype with increased expression of cytokines IL-6 and IL-1β [62]. C9ORF72-knockout mice lacking the expression of C9ORF72 in MNs, however, do not develop MN degeneration or disease. It seems that the expression of C9ORF72 in innate immune cells, including macrophages and microglia, is not sufficient to cause MND in a mouse model, unless C9ORF72 is also expressed in MNs.

Impaired regulation of autophagy and enhanced inflammation can be caused not only by mutations in *C9ORF72*, but also in other genes, e.g., *OPTN*, *TBK1*, *SQSTM*, or *VCP*. The inhibition of autophagy in mSOD1^G93A^ MNs in transgenic mice accelerated the disease onset, but prolonged lifespan [179]. However, autophagy inhibition in glia might have different effects than in neurons. Additionally, the responses may vary among cell types. For more details, see the review article [180].

### 7.3. Microglia in TDP-43 Pathology

In addition to other animal models, transgenic TDP-43^Q331K^ mice also show increased microglial activation and MN degeneration [162]. In more than 90% of ALS patients, cytoplasmic TDP-43 aggregates that accumulated in spinal cord were observed post mortem [29]. The translocation of TDP-43 from nucleus to cytoplasm is thought to be a part of the ALS pathogenesis [181], as the aggregates are strong triggers of microglia immune responses. The NLRP3 inflammasome is an important part of the innate immune system activated by protein aggregates—an intracellular signaling complex [182,183,184,185]. The activation of this complex requires a priming signal and it results in the upregulation of NLRP3 and cytokine precursors: pro-IL-1β and pro-IL-18. This process is followed by an activation step, which involves the recruitment of the inflammasome adapter (apoptosis-associated protein containing a caspase recruitment domain), activation of the caspase-1 protease, and, finally, the cleavage and release of IL-1β and IL-18 [186,187]. Deora, V. introduced the first report of increased inflammasome gene expression in TDP-43^Q331K^ mice [187]. Their study also demonstrates that TDP-43 proteins (as well as SOD1) induce inflammasome activation in primary microglia.

TDP-43 is able to induce microglial activation via interaction with the CD14 receptor on the cell surface and initiating a proinflammatory cascade and neuronal cytotoxicity. When influenced by activated TDP-43, microglia start to express NOX2 and produce TNF-α and IL-1. Microglia that are activated by TDP-25 cause the death of MNs when co-cultured together, but TDP-25 and TDP-43 do not seem to be toxic to neurons on their own, only when microglia are present [188]. These results are consistent with the previous work of these authors, where they demonstrated these results in mSOD1^G93A^ mice [189].

Interestingly, there are also studies showing that microglia in the environment of MNs can even have a positive impact on their survival [190]. Researchers using the mSOD1 model usually propose the elimination of microglia as a beneficial strategy [176]. Nevertheless, Spiller and colleagues suggested that, in TDP-43 pathology (using rNLS8 mutation), it might be more efficient to find ways of how to encourage appropriate microglia-mediated inflammation, to clear pathological TDP-43 proteins and help axonal regeneration during the onset/progress of ALS [190].

In our point of view, the Spiller suggestion for TDP-43 pathology seems to be promising. However, as they pointed out, their model, unlike mSOD1, has wild-type microglia and intact BBB. Based on this fact, we hypothesize that the possible modulation of microglia towards their anti-inflammatory state could be more beneficial in SOD1 and C9ORF72 mutants. In a recent study [191] while using SOD1^G93A^, they ascribe the neuroprotective effect to the shift of microglia from their pro- to anti-inflammatory state and similarly, study using C9ORF72 mutant carriers [192] showed that immunotherapy can inhibit the activation of microglia and, thus, prevent the disease onset. Overall, it seems that the ability to modulate the character of microglia could make an impact on the onset/progress of ALS whether it is the shift to their neuroprotective state or encouragement of appropriate microgliosis.

## 8. NG2 Glia

The adult CNS contains a widely distributed population of oligodendrocyte progenitor cells (OPCs), which are also called NG2 glia. These cells have the capacity to replace injured cells or those lost due to age-related degeneration. They differentiate not only into myelinating oligodendrocytes, but also into protoplasmic astrocytes, which are specifically localized in the gray matter [193]. It has also been suggested that NG2 cells can differentiate into neurons, which might possibly happen in the different brain regions. Two groups described NG2 cell ability to give rise to pyramidal-like neurons in dorsal and ventral forebrain cortext [194,195]. Alternatively, Aguirre and colleagues showed that they express neuronal markers in the subventricular zone or differentiate into GABAergic interneurons in the hippocampus [196].

Following CNS injury, NG2 glia start to proliferate extensively and their differentiation into oligodendrocytes is enhanced [100,197]. However, following neurodegeneration, NG2 glia only give rise to oligodendrocytes [197], while their rate of proliferation remains high during the whole progress of the disease [100]. The proliferation of NG2 cells in mSOD1 murine models differs among brain regions and it is more prominent in the ventral gray matter, where the proliferation increases before the onset of disease symptoms and continues to rise during ALS progression. Interestingly, NG2 glia of ventral white- and dorsal gray matter display a lower proliferation rate, which occurs in the later stages of ALS progression [100]. When compared to NG2 cells in healthy white matter, the number of NG2 cells in TDP-43 mutant mice is markedly increased and they have a different morphology, which is characterized by enlarged cellular soma and lesser processes [198]. Overall, there is little information regarding NG2 glial contribution to ALS pathology, therefore more information should be obtained, and more research undertaken to understand their role in this disease.

## 9. Oligodendrocytes

Oligodendrocytes in the CNS are responsible for the myelination of axons. Myelin plays an important role, not only in the electrical insulation of the axons, but also in their provision with trophic and metabolic support [199]. Oligodendrocytes and myelinated axons are metabolically coupled. Oligodendrocytes are able to transform glucose to lactate or pyruvate and provide it to neurons via monocarboxylic acid transporters (MCT1, MCT2) [200,201]. N-methyl-D-aspartate (NMDA) receptors conduct the regulation of this process, which are present on oligodendrocytes and are able to recognize neuronal activity, incorporate additional glucose transporters, and thus increase the import of glucose [202]. Oligodendrocytes and myelin are not only central to the pathological mechanisms of inflammatory diseases (e.g., multiple sclerosis), but they may also play an important role in several neurodegenerative diseases, such as ALS The importance of oligodendrocytes in disease onset/progression was suggested in SOD1-, C9ORF72-, and TDP-43 pathologies.

### 9.1. Oligodendrocytes in SOD1 Pathology

Oligodendrocytes are strikingly affected during ALS and their degeneration seems to precede MN death in the mSOD1^G93A^ mouse model [100,203]. Additionally, a rapid proliferation of oligodendrocyte progenitors has been shown in the spinal cord of SOD1^G93A^ mice; however, newly derived cells failed to mature and replace degenerated oligodendrocytes. Consequently, axons of MNs remained demyelinated [100,203]. It was demonstrated that the reduction in mSOD1^G93A^ synthesis in oligodendrocytes, which occurs during their early maturation, produces a more substantial delay in the disease onset [100] than that observed in MNs in mSOD1^G37A^ [99,204]. Oligodendrocytes support MNs by providing the direct supply of the energy metabolite lactate to axons due to the action of MCT1, as mentioned above. In the ALS mouse model, the mSOD1^G93A^ impairs the expression of MCT1 in oligodendrocytes and a similar reduction in MCT1 accumulation can be found in sALS [200].

In the gray matter of the spinal cord of SOD1^G93A^ ALS mice, mature oligodendrocytes extensively degenerate, prior to the onset of disease symptoms [100,203]. The selective deletion of mSOD1^G93A^ from oligodendrocytes markedly delays the onset and prolongs the survival of mice [100]. In vitro studies showed that oligodendrocytes that are obtained from ALS patients are able to induce MN death when co-cultured together [205]. These findings suggest that the dysfunction of mature oligodendroglia that is caused by mSOD1 can have a critical role in the ALS.

One of the studies of mature oligodendrocytes in SOD1 pathology was carried out while using a zebrafish model [206], in which the mSOD1^G93A^ was only selectively expressed in oligodendrocytes. They found that mSOD1 directly induces the degeneration of oligodendroglia, via the disruption of myelin sheaths as well as the downregulation of MCT1, which resulted in the degeneration of the spinal cord MNs. The dysfunction of oligodendrocytes was also associated with behavioural abnormalities, learning impairments, and motor defects in the early symptomatic stage of ALS. In addition, treating the fish with K^+^ channel inhibitors rescued abnormalities in behaviour, but, unfortunately, without rescuing the expression of MCT1. These results suggest that myelin disruption induces abnormalities in behaviour, independently of MCT1. Overall, the dysfunction of mature oligodendrocytes is presumably sufficient to induce MN degeneration [206].

It was also shown that oligodendrocytes expressing mSOD1^G93A^ are able to induce electrophysiological changes in wild type MNs, ultimately leading to MN death [205]. These data are consistent with the findings of Pieri and colleagues [207], who reported that increased persistent Na^+^ currents are selectively altered and cause hyperexcitability. Data from both mentioned studies [205,207], while using an in vitro approach or mouse model, confirmed that cortical hyperexcitability happens to be one of the first neuronal alterations detected in patients with ALS [208] before the symptom onset [209]. Marcuzzo and colleagues, who worked with cultured neurons from SOD1^G93A^ mice aged one day, also just recently confirmed this [210].

### 9.2. Oligodendrocytes in C9ORF72 and TDP-43 Pathology

TDP-43 and FUS-positive inclusions were found in oligodendrocytes of ALS patients in post mortem analyzed tissue [211], and their presence suggests an impairment of autophagy in oligodendrocytes. A study comparing both human and mouse ALS oligodendrocytes was performed since it is not obvious whether observations obtained in mouse models of ALS hold true in a wide spectrum of ALS patients [205]. The in vitro study showed that oligodendroglia successfully differentiate from mouse neural progenitor cells (NPCs) and human iPSCs, and neural progenitor cells iNPCs [130], from both ALS- and non-ALS samples. According to these results, the ability of ALS oligodendrocytes to pass the toxicity on MNs in vitro does not depend on their origin. The reduction of mSOD1 in OPC can rescue the toxicity, but the reduction of mSOD1 in differentiated oligodendrocytes does not have the same effect. The toxicity of cells carrying *C9ORF72* repeat expansion shows no response to the reduction of SOD1 suggesting their SOD1 independence [205]. Moreover, they also did not display dysfunction in lactate release. These findings suggest that this mutation defines a specific subgroup of patients with ALS within a neuropathological spectrum and that might not respond to the same treatment than those carrying mSOD1.

TDP-43 aggregates were found in oligodendrocytes and in microglia. TDP-43 seems to be necessary, in a cell-autonomous manner, for the correct function of mature oligodendrocytes. The depletion of TDP-43 causes the RIPK1-mediated necroptosis of oligodendroglia and the down-regulation of proteins, which are essential for myelination, but exhibits no apparent toxicity on MNs. There also seems to be an inner difference between the regeneration in white and gray matter. NG2-positive oligodendrocyte precursors in the white matter were able to compensate the loss of mature oligodendrocytes, unlike NG2 glia in the gray matter [198].

## 10. Pericytes

Although pericytes are not glial cells, they have been included in this review, because they have common characteristics with glial cells, such as activation, proliferation, and migration into the injured area, where they are involved in the “glia” scar formation. They form a part of neurovascular unit (NVU), which also includes astrocytes, endothelial cells, and neurons. The cells forming NVU, together with extracellular components, form a complex regulating cerebral blood flow and nutrient delivery [212]. Therefore, it is evident that pericytes are in close contact with other cells of the CNS, and they are likely to affect glial cell functions. The ALS pathology as well as the pathology of other neurodegenerative diseases, is the result of complex events in various cell types of the nervous system and intercellular interactions. Therefore, it is very important to look at the pathology of these diseases in a comprehensive way to understand how changes in the function of individual cell types can affect the behavior of other cells in the CNS.

Pericytes are a special type of mural cells, which are cells that wrap the endothelium and contribute to the forming of BBB and blood-spinal cord barrier (BSCB). Although their identification is complicated due to the heterogeneity of this cellular type (for review see [213]), the main characteristics are as follows. The name “pericytes” refers to their localization enwrapping the endothelium of brain microvessels, e.g., capillaries, post-capillary venules, and terminal arterioles [214]. The main morphological features of pericytes are the spatially isolated nuclei and processes that extend along the capillary. The most often used markers are platelet-derived growth factor receptor (PDGFRβ), alanyl aminopeptidase (CD13), neural-glial antigen 2 (NG2), and desmin [215]. Pericytes play important roles in many functions in the CNS, such as Ca^2+^-dependent constriction/dilation of capillaries that are surrounded by pericytic processes, through membrane depolarization or neurotransmitter receptor activation [216] and the formation of new blood vessels. The two-way interaction between endothelium and pericytes includes a production of platelet-derived growth factor-BB (PDGF-BB) by endothelial cells and its binding on pericytic PDGFRβ, which results in pericyte proliferation and migration into the endothelial tube. On the other hand, pericytes release angiopoietin-1 (Ang-1), which, by binding to endothelial tyrosine kinase receptors Tie-2, activates the proliferation of endothelial cells and the subsequent formation of new blood vessels (for review see [217]). Finally, the presence of pericytes around brain capillaries is also indispensable to the BBB maintenance. In the case of pericyte loss, a severe disruption of BBB occurs, which results in the entry of large molecules and toxic substances into the brain parenchyma [218]. Accordingly, pericytes are involved in the *regulation of immune cell entry* into the brain parenchyma. The pericyte loss results in the increased entry of leukocytes into the brain and it also modulates the inflammatory response [219,220]. Interestingly, several investigators recently showed that a special type of pericytes might proliferate and migrate in response to the CNS injury and, thus, contribute to the “glia” scar formation [221,222].

There is currently not much information regarding the role of pericytes in ALS, but their complex role in CNS functioning suggests that they might participate in the pathophysiological processes of this disease. In general, the roles of pericytes in CNS pathology mainly include disturbances of the above-mentioned functions—BBB maintenance, angiogenesis, controlling the capillary diameter, and scar formation. In connection with ALS, the loss of pericytes and subsequent disruption of the BSCB have been studied. BBB and BSCB damage was found in patients with ALS, either on the basis of albumin CSF/serum ratio in the cerebrospinal fluid (CSF) [223,224] or while using post mortem pathological analyzes [225,226]. Similarly, a spontaneous BSCB breakdown, as a major cause of MN death, has also been described in SOD1 mutant models of ALS—G93A, G37R, and G85R [227,228,229,230,231]. Winkler and co-authors have addressed the question of the pericytic role in the BSCB disruption [232]. They showed that, in human ALS patients (sALS and fALS), the capillary leakage of erythrocytes and plasma proteins, which results from vascular rupture, coincides with the reduction in pericyte population. Additionally, Sasaki and co-authors suggested that disturbed pericytes may cause capillary constriction and subsequent reduction of microcirculation, resulting in MN degeneration, based on their structural and morphological analyses of human spinal cord tissue post mortem samples [233]. In another study, these authors observed similar results in conditional TDP-43 knockout mice [234]. They demonstrated that the loss of TDP-43 protein might lead to BSCB disruption, which in turn contributes to the MN degeneration. Finally, and interestingly, the intraperitoneal application of pericytes led to the prolonged survival of SOD1^G93A^ male mutants and, when co-cultured with human iPSC derived MNs and other neuronal cells, pericytes induced the expression of antioxidant enzymes SOD1 and catalase [235].

## 11. Crosstalk of Glial Cells

As mentioned above, all of the glial cells in ALS undergo a series of changes and become nonprofitable to MNs, which in the end leads to the neurodegeneration. ALS is a complex and multifactorial disease, so it seems likely that the crosstalk of different cell types contributes to trigger the neurodegeneration rather than just one cell type. Reactive astrocytes can secrete pro-inflammatory mediators and, thus, affect the activation of microglia, which are known to contribute to the disease progression when activated. In the mSOD1 model, removing of the mSOD1 from astrocytes postponed the activation of microglia and, thus, extended the survival [101,236]. The overexpression of astrocytic TGF-β1 in mSOD1, on the other hand, seems to worsen the progression due to interference with microglial neuroprotective function [237]. The other way around, activated neuroinflammatory microglia can induce neurotoxic reactive astrocytes [106], which appear in various neurodegenerative diseases, including ALS. Astrocytes could possibly affect also oligodendrocytes as they create an environment that promotes OPC recruitment, migration and differentiation [238]. In the adult rat spinal cord, after demyelinating injury were the OPCs unable to remyelinate axons in the absence of astrocytes, even though their recruitment was successful [239]. This suggests that changes of astrocytes may affect the impaired regeneration of oligodendrocytes that were observed in ALS. These results support the idea of glial cell crosstalk playing an important role in ALS pathology, but we are convinced that conducting more studies is necessary for better understanding the glia cooperation and their complex role should be then taken into the account when developing new therapeutic approaches.

## 12. Glia-Oriented Preclinical Studies

Based on preclinical testing, dozens of drugs have been claimed as promising for ALS treatment during the past few years. However, significant difficulties were met during translating these findings toward patients with ALS, regardless of the interesting results obtained in basic research. Large number of newly discovered compounds failed when tested in clinical trials with ALS patients and such negative outcome is possibly due to the vast ALS complexity. Here, we aim to focus on glial cells, namely astrocytes and microglia, as they were identified as possible triggers of the disease or they were suggested to play an important role in ALS progression. The glia-oriented preclinical studies can be roughly divided into three categories that are based on their therapeutic targets. They encompass astrocytic glutamate uptake, development of astrogliosis and microglia activation, especially the appearance of proinflammatory phenotype of microglia.

### 12.1. Increasing Glutamate Transporter Levels

In the first group, numerous studies focused on glutamate transporter EAAT2 (GLT-1) expression in glia as a key event, which might lead to excessive glutamate receptor activation in MNs and result in their death. Lapucci et al. [240] turned the attention to histone deacetylases (HDACs), as their inhibition gave promising results in the treatment of different neoplasms and primary muscular diseases. HDACs belong to epigenetic enzymes that are responsible for the de-acetylation of different proteins that are involved in transcriptional regulation and their pharmacological targeting affects gene expression profiles. Previous studies employing non-class selective HDAC inhibitors revealed that they lessen ALS development in mice, but they fail when translated to ALS patients, presumably due to lack of class selectivity [241]. Lapucci and colleagues investigated the effect of the Class II HDAC inhibitor MC1568 on expression of EAAT2, glutamate uptake, and survival of SOD1^G93A^ ALS mice [240]. They reported that MC1568 increased EAAT2 expression in primary astrocyte cultures, but such elevation did not lead to increased glutamate uptake. However, when tested in SOD1^G93A^ mice, the daily application of MC1568 (intraperitoneally) gave decreased expression of EAAT2, as well as glutamate uptake in spinal cord back to control levels.

Another approach for increasing EAAT2 expression comprises the use of compounds that may increase EAAT2 expression through translational activation, such as a pyridazine derivative LDN/OSU-0212320, which was tested in the SOD1^G93A^ mouse model of ALS [242]. Mice received LDN/OSU-0212320 at 84 days of age (six times per week, at the same time each day) until death or until the brain tissues were isolated at 120 days of age. The authors showed that LDN/OSU-0212320 increases EAAT2 expression through translational activation in a primary astrocyte cell line, it protected cultured neurons from glutamate-mediated excitotoxic injury and death via EAAT2 activation and it markedly delayed motor function decline and extended the lifespan in SOD1^G93A^ mice. Additionally, their study revealed that LDN/OSU-0212320 treatment results in the activation of protein kinase C and subsequent Y-box–binding protein 1 (YB-1) activation, which regulates the activation of EAAT2 translation. Their results suggest that the use of small molecules for enhancing EAAT2 translation may be a beneficial therapeutic strategy for ALS.

Similarly, many b-lactam antibiotics, such as ceftriaxone, were shown to be potent stimulators of GLT-1 expression. Cefriaxone action seems to be mediated through increased transcription of the GLT-1 gene7. Rothstein and colleagues [243] treated SOD1^G93A^ mice daily with ceftriaxone starting at 12 weeks of age. When delivered to animals, the ceftriaxone increased both the brain expression of GLT-1 (astrocytic) and its biochemical and functional activity. Ceftriaxone was neuroprotective in vitro when used in models of ischemic injury and MN degeneration, both being based, in part, on glutamate toxicity [244]. When used in the animal model of the fatal disease ALS, the drug delayed loss of MNs and muscle strength, and increased mouse survival. Thus, these studies provide a class of potential neurotherapeutics that act to modulate the expression of glutamate neurotransmitter transporters via gene activation. Of note, a clinical trial with ceftriaxone in ALS has been prematurely stopped, because of the lack of therapeutic effect in ALS patients. Despite promising data from stage two [245], stage three of this trial of ceftriaxone in ALS did not show clinical efficacy [246].

Ganel and co-authors undertook an interesting strategy [247], who selectively up-regulated the glial Na^+^-dependent glutamate transporter GLT-1 by the aneuroimmunophilin ligand. FK506-binding protein (FKBP) immunophilins are ubiquitous cytosolic proteins, which are concentrated in neural tissue (also termed neuroimmunophilins) and may increase the expression level of GLT-1. Their synthetic counterpart, GPI-1046, is a non-immunosuppressive derivative of FK506 that displays neuroprotective and neuroregenerative actions in several systems [248,249]. In the study of Ganel et al. (2006) employing mouse model of ALS, it induced the selective expression of GLT-1 in vitro and in vivo, which was associated with a marked increase in dihydrokainite sensitive Na^+^-dependent glutamate transport protecting MNs in an in vitro model of chronic excitotoxicity and prolonging the survival of SOD1^G93A^ mice. These studies suggest that neuroimmunophilins can regulate GLT-1 and their ligands could serve as therapies for neurodegenerative disorders.

The attention was also turned to anthocyanins—flavonoid compounds derived from fruits and vegetables that possess antioxidant, anti-inflammatory, and antiapoptotic actions. Especially callistephin, which is a primary anthocyanin constituent, displays significant neuroprotective effects against glutamate excitotoxicity and mitochondrial oxidative stress in vitro in primary cerebellar granule neurons. Winter and coauthors [250] tested an anthocyanin-enriched extract from strawberries (SAE) in SOD1^G93A^ mice. Interestingly, mice that were supplemented with SAE experienced a marked delay in disease onset and a statistically significant extension in survival in comparison to their untreated mutant counterparts. SAE treated SOD1^G93A^ mice significantly preserved the grip strength throughout disease progression. Moreover, histopathological analysis demonstrated that astrogliosis in the spinal cord is reduced.

### 12.2. Targeting Activation of Astrocytes and Microglia

Anti-oxidative agents were tested as a possible way of treating ALS since oxidative stress plays a crucial role in the progression of MN loss observed in ALS. In 2011, bromocriptine (BRC) methylate, the dopamine D2 receptor agonist, was subjected to preclinical testing in SOD1^H46R^ mice [251]. The reason for selecting BRS was its identification as an upregulating compound of neuronal apoptosis inhibitory protein (NAIP). The administration of BRC after the onset of symptoms remarkably sustained the motor performance and prolonged 12% of the post-onset survival interval of the mice, suppressed MN loss in the spinal cord, significantly reduced activation of astrocytes, reduced the levels of inflammatory factors, inducible nitric oxide synthase (iNOS) and tumor necrosis factor (TNF)-α, and oxidative damage. The authors also demonstrated that, in mouse primary astrocyte cultures, the release of TNF-α after lipopolysacharide (LPS) exposure was reduced by the BRC treatment. Further, in vitro studies using SH-SY5Y cells suggested that the neuroprotective efficacy of BRC in ALS (SOD1^H46R^) mice attributed, at least in part, to the upregulation of several anti-oxidative-stress genes, nuclear factor related erythroid 2-related factor 2 (Nrf2), activating transcription factor 3 (ATF3), and heme oxygenase-1 (HO-1), and mediated effective synthesis of glutathione (GSH), presumably in astrocytes.

Phytocannabinoids, but also synthetic cannabinoids and even the signaling lipids, which are part of the so-called endogenous cannabinoid system, have been demonstrated to possess important neuroprotective properties. Therefore, neuroprotective effects of the cannabigerol quinone derivative VCE-003.2 were tested in SOD1^G93A^ transgenic mice [252]. It acts as a neuroprotectant by activating the peroxisome proliferator-activated receptors (PPARs), a group of nuclear receptor proteins, which function as transcription factors that regulate the expression of genes [253]. PPARs play essential roles in the regulation of cellular differentiation, development, and metabolism (carbohydrate, lipid, protein), and tumorigenesis in higher organisms. The administration of VCE-003.2 (intraperitoneally, 10 mg/kg) improved most of the SOD1^G93A^ model neuropathological signs. It attenuated the weight loss and the anomalies in neurological parameters, preserved spinal cholinergic motor neurons, and reduced astroglial reactivity. Furthermore, it reduced the LPS-induced generation of TNF-α and IL-1β in cultured astrocytes that were isolated from SOD1^G93A^ transgenic newborns.

Chemokine receptors CXCR4, and their ligand CXCL12, also known as stromal-cell-derived factor (SDF1), might represent suitable targets for developing new ALS therapy. They are able to modulate both neuronal function and apoptosis by glutamate release signaling and rapidly mobilize hematopoietic stem and progenitor cells (HSPCs) from the bone marrow into the blood of mice. The inhibition approaches towards the CXCR4/CXCL12 signaling may thus result in preventing neuronal apoptosis and alter the HSPCs migration and their homing. Such inhibition can be achieved by means of treatment with AMD3100, which is an antagonist of the chemokine receptor CXCR4. In the study of Rabinovitch-Nikitin et al. [254], SOD1^G93A^ mice were treated until the end stage of the disease. Chronic administration of AMD3100 led to significant extension in mouse lifespan and improved the motor function and weight loss. Noteworthy, this approach significantly improved microglial pathology and decreased the levels of pro-inflammatory cytokines in spinal cords of treated SOD1^G93A^ mice. Furthermore, AMD3100 treatment decreased BSCB permeability by increasing the tight junction proteins levels and amplified the MN count in the lamina X, area of the spinal cord, where adult stem cells are formed.

In the same model of ALS, SOD1^G93A^ mice, contribution of colony stimulating factor 1 receptor (CSF1R) signaling to inflammation was studied, as this pathway was previously reported to control the expansion and activation of microglial cells. Martinez Muriana et al. [255] described that microglial cell proliferation in the spinal cord of SOD1^G93A^ mice correlates with the expression of CSF1R and its ligand CSF1. The administration of GW2580, which is a selective CSF1R inhibitor, results in reduced microglial cell proliferation in SOD1^G93A^ mice and indicates the importance of CSF1-CSF1R signaling in microgliosis in ALS. Moreover, GW2580 treatment slowed disease progression, attenuated MN cell death, and extended survival of SOD1^G93A^ mice. GW2580 treatment also protected skeletal muscle from denervation prior to its effects on microglial cells. Their findings suggest that the blockage of CSF1R signaling might represent a suitable approach for attenuating inflammation in ALS.

A recent study on ALS mouse model has proven that IGF-1 might be a promising therapeutic drug as well. Hu and colleagues [256] injected SOD1^G93A^ mice with self-complementary adeno-associated virus serum type 9 encoding the human IGF-1 (scAAV9–hIGF1, i.t.). The delivery of scAAV9–hIGF1 to the subarachnoid space of presymptomatic and symptomatic ALS mice resulted in hIGF1 protein expression all over the brain and spinal cord. It decreased MN loss, improved motor function, and significantly extended a life span. In addition, the overexpression of hIGF1 down-regulated the levels of iNOS, TNF-α, and PP65 in the lumbar spinal cord of SOD1^G93A^ mice. Knocking down mIGF1 via the CRISPR/Cas9 system confirmed that the levels of iNOS, TNF-α, and PP65 are increased in the lumbar spinal cord of ALS mice Taken together, these data indicated that IGF1-mediated suppression of NF-κB activation in microglia is a novel molecular mechanism that underlie MN death in ALS.

Similarly to IGF-1, the use of cyclic nitroxides, such as tempol, might provide neuroprotection and improve lifespan. These drugs are multifunctional antioxidants and present low toxicity in vitro and in vivo. Tempol (4-hydroxy-TEMPO) is considered to be a cyclic nitroxide with low molecular weight and excellent cellular permeability. Although tempol is mainly characterized as the antioxidant, other effects in different pathological conditions were demonstrated, including anti-apoptotic, antiinflammatory, immunomodulatory, and therapeutic properties. It was also established that tempol restores muscular force in normal and dystrophic animals, which demonstrated that it can be considered a candidate for the treatment of neurodegenerative diseases, such as ALS [257,258].

Using AAV9 vectors, the SOD1^G93A^ mice were treated with tempol, which promoted greater neuronal survival (23%) at initial stage of symptoms compared to untreated mice [259]. The intense reactivity of astrocytes and microglia that was observed in vehicle animals was significantly reduced in the lumbar spinal cords of SOD1^G93A^ mice treated with tempol. In addition, the groups treated with tempol showed reduced expression of proinflammatory cytokines (IL1β and TNFα) and a three-fold decrease in the expression of TGFβ1 at initial stage of symptoms when compared with the group treated with vehicle. Altogether, the results indicate that treatment with tempol has beneficial effects, which delays the onset of the disease by enhancing the neuronal survival and decreasing glial cell reactivity during ALS progression in SOD1^G93A^ mice.

An interesting approach was recently published, employing an active poly-GA vaccination, which prevented microglia activation and motor deficits in a C9ORF72 mouse model of ALS [192,260]. Non-canonical translation of the expanded repeats in C9ORF72 resulted in abundant poly-GA inclusion pathology throughout the CNS. Such (GA)149-CFP expression triggers motor deficits and neuroinflammation in mice, and, as poly-GA is transmitted between cells, the therapeutic potential of anti-GA antibodies was tested. Poly-GA vaccinated mice showed less poly-GA aggregation and cytoplasmic mislocalization of TDP-43, and more importantly, vaccination with Ova-(GA)10 largely prevented motor symptoms and microglia/macrophage activation in vivo.

Recently, histamine has been postulated to have a key regulatory role in experimental autoimmune encephalitis and multiple sclerosis pathogenesis. As a mediator of inflammation and immune responses, histamine induces the release of pro-inflammatory factors, such as TNF-α and IL-6 from activated microglia, via H1R and H4 receptor-MAPK and PI3K/AKT-NF-kappa B signaling pathway. Moreover, it induces the production of reactive oxygen species and also acts as a nitric-oxide regulating factor by stimulating inducible nitric oxide synthase expression in the microglia from neonatal rat brain. Therefore, pharmacological targeting of histamine receptors appears to be an appropriate disease modifying therapeutic approach against neuroinflammatory diseases, including ALS. Clemastine, which is also known as meclastin, is an ethanolamine-derivative, first generation histamine H1 antagonist with anticholinergic properties and sedative side effects, was tested in SOD1^G93A^ mice [261]. The authors demonstrated that chronic clemastine administration reduces microgliosis, modulates microglia-related inflammatory genes, and enhances MN survival. Moreover, in vitro clemastine is able to modify several activation parameters of SOD1^G93A^ microglia and, particularly, CD68 and arginase-1 expression, as well as phospho-ERK1/2 (p-ERK1/2) and NOX2 levels. These results strongly encourage its further use as a candidate for preclinical trials and a tool for discerning neuroinflammation in ALS since clemastine is already used in clinical practice.

### 12.3. Steroids

Neuroactive steroids are already available for the treatment of various CNS diseases: progesterone (PROG) recovers myelin after damage [262], prevents neuronal loss after brain trauma, enhances motor function, and reduces total infarct volumes after stroke [263]. In addition, improves neuronal mitochondrial function in neurodegenerative diseases [264], enhances learning and memory [265], and shows protective effects in diabetic peripheral neuropathy, epilepsy, Huntington’s disease, Alzheimer’s disease, multiple sclerosis, and Parkinsonian models [266]. The study of Gargiulo-Monachelli and co-authors [267] compares the effects of PROG and of synthetic progestin norethindrone (NOR) treatment on neuronal, inflammatory, and clinical parameters in the spinal cord of Wobbler (Wr) mice. At the age of two months, the mice were subcutaneously implanted with a pellet of PROG or NOR. The administration of PROG to Wr mice for periods from three weeks to two months attenuated neuropathology, inhibited oxidative stress, enhanced the expression of genes that are involved in MN function, increased survival, and restored axonal transport. Untreated Wr mice showed typical clinical and spinal cord abnormalities that were normalized in those with PROG treatment. Surprisingly, the authors found that NOR does not increase the immunoreactivity and gene expression of choline-acetyltransferase in MNs. Based on GFAP immunoreactivity, NOR markedly decreased astrogliosis, favored proinflammatory mediators, promoted the inflammatory phenotype of IBA1^+^ microglia, increased mRNA level of the receptor for advanced glycation end products (RAGE), and protein expression and the activity of nitric oxide synthase (NOS)/NADPH diaphorase in the cervical spinal cord. Additionally, NOR treatment produced atrophy of the thymus. The combined negative NOR effects on clinical assessments (forelimb atrophy and rotarod performance) suggest a detrimental effect on muscle tropism and motor function.

Another study [268] employing SOD1^G93A^ mice concentrated on the role (beneficial or detrimental) of physical exercise and the use of anabolic steroid, namely treatment with the anabolic androgenic steroid 19-nortestosterone (nandrolone). During daytime, the mice were subjected to physical exercise, comprising running on a custom-made treadmill at a velocity and nandrolone was injected once a week. Nandrolone treatment markedly enhanced MN loss, and this detrimental effect was reverted by the combination with exercise, which resulted in increased MN survival. Astrocytic activation was markedly increased after nandrolone treatment when MN damage was most severe, while microglia activation was most noticable after physical exercise when MN damage was less severe. These results indicate a vulnerability of mSOD1 MNs to nandrolone treatment, a potential neuroprotective effect of physical exercise, and a modulation by glial cells in the ALS murine model.

Finally, angiogenesis got to the center of interest in a recent study, which focused on the role of systematically delivered angiogenin in the SOD1^G93A^ mouse model [269]. Administration of human angiogenin (ribonuclease 5)—huANG—led to its elevation in serum. huANG was taken up by astrocytes and endothelial cells and it led to increased survival and delayed motor dysfunction. The authors suggest that huANG represents a new class of pleiotropic ALS therapeutic that acts on the spinal cord vasculature and glia (presumably on astrocytes) to delay MN degeneration and disease progression.

## 13. Current Treatment and Clinical Trials

Over the past 20 years, there has been limited success in the development of effective treatment for ALS. The incorporation of new treatment options has proven difficult, which is mainly due to the complex nature of this disease. Although a variety of compounds with different mechanisms of action were examined, the vast majority of these compounds failed to show clinical efficacy in human ALS trials. Currently, Riluzole, together with Edaravone, are the only available treatments for ALS, and these only bring mild benefits in terms of survival and quality of life. Recently, Masitinib has been shown to slow the progression of the disease, provided the treatment is started in the early stages, and it is likely to be approved by the FDA (Food and Drug Administration) in the near future.

Several measurements, both specific and non-specific for ALS, are designed for the evaluation of clinical efficacy of a specific compound. These include: AALS (Appel ALS Rating Scale), ALSAQ-5 (ALS Assessment Questionnaire—five item), ALSAQ-40 (ALS Assessment Questionnaire—40 item), ALSFRS-R (ALS Functional Rating Scale—Revised), CAFS (Combined Assessment of Function and Survival), ECAS (Edinburgh Cognitive and Behavioural ALS Screen), FVC (Forced Vital Capacity), MMT (Manual Muscle Testing), MRC (Medical Research Council scale), MVIC (Maximum Voluntary Isometric Contraction), and SVC (Slow Vital Capacity). An important rating of ALS progression is also the survival rate; this is usually described as the time to death, tracheostomy, or permanently assisted ventilation. Each study has specific endpoints that describe the goal of that study and effectiveness of the provided treatment.

### 13.1. Riluzole

Riluzole was the first compound approved and, for more than 20 years, it was the only drug available for the treatment of ALS. Riluzole got its approval in 1995 based on the results of two studies that were conducted in the 1990′s, which demonstrated the safety and tolerability of this drug, as well as the prolonged survival of patients [270,271]. A Phase III trial later failed to demonstrate any clinical efficacy [272]. Although Riluzole was the first described to have an anti-glutamergic effect, the mechanism of its action was never fully understood (for more details see [273]).

### 13.2. Edaravone

Edaravone (sold under names Radicava and Radicut) is a novel anti-oxidative agent that is used for the treatment of ALS. It is believed to be a free-radical scavenger [274], although the precise mechanism of its action is not fully elucidated.

The first study exploring the effects of Edaravone in 19 ALS patients used an open-label design. Even though this study used an alternative design, it was successful in its primary endpoint (a statistically significant improvement of ALSFRS-R slope).

Three confirmatory trials were launched to further evaluate the clinical effect of Edaravone [275]. The first confirmatory study with broad inclusion criteria contained the largest number of patients (206, with 102 on Edaravone and 104 on placebo) with ALSFRS-R as the primary endpoint. However, this study was unsuccessful in meeting this endpoint. A post-hoc subgroup analysis showed a trend of a reducing ALSFRS-R slope decline in patients with moderately severe disease progression [276]. The second confirmatory study narrowed the inclusion criteria (FVC > 60%, disease onset < 3 years) and only included a small number of patients—25. This study also failed in its primary endpoint (ALSFRS-R scale) [277]. The third confirmatory study further narrowed the inclusion criteria (FVC > 80%; disease onset < 2 years) and excluded patients with the most and least severe disease progression. This trial assessed 137 patients (69 Edaravone, 68 placebo) for a period of 24 weeks and succeeded in achieving its primary endpoint (ALSFRS-R scale) and one of the eight secondary endpoints (ALSAQ-40 questionnaire) [278]. Of the 137 patients, 124 continued the study for a 24-week extension period. This extension reported no significant differences in any of its endpoints (ALSFRS-R, FVC, and ALSAQ-40), neither for the 24-week period nor for the 48 weeks combined [279]. Edaravone was approved for use in Japan based on the results from the third confirmatory study (in June 2015), and up to this date (October 2019) also in South Korea, USA, Canada, Switzerland, and Brazil.

It is important to point out that all of the trials mentioned above were conducted in the non-representative Japanese population. It remains to be seen whether Edaravone will also achieve the same clinical efficacy in other populations, as only one study with non-Japanese patients reported results in a peer-reviewed journal [280]. This study included 31 ALS patients from Northern Italy when compared to 50 historical controls (late ALS patients not treated with Edaravone who met the same exclusion and inclusion criteria) and compared the changes in ALSFRS-R scale, FVC score, and MRC score. This study reported no statistical significance in any of its endpoints. Another study currently evaluates the efficacy of Edaravone in 20 Iranian patients (trial ID#: NCT03272802) with MMT, ALSFRS-R, and ALSAQ-40 being its endpoints. Additionally, an observational study will be conducted to assess differences in the levels of ALS biomarkers between the patients treated and not treated with Edaravone (trial ID#: NCT04097158).

These two current FDA-approved drugs for ALS only modestly attenuate disease progression. Both of these small molecule drugs are based on a single mechanism of action, while ALS is a multifactorial disease and therapeutic approaches should take the multiplicity of mechanisms that underlie motor neuron degeneration in this disease into account.

### 13.3. Masitinib

Masitinib is a highly specific tyrosine kinase inhibitor (developed by AB Science) that blocks the CSF1R and c-Kit pathways. It blocks the activation of immune cells like mast cells and macrophages, which have been shown to play a role in MN degeneration in SOD1^G93A^ rat model [281,282]. Masitinib has been shown to reduce neuroinflammation, microgliosis and prevent damage to neurons [283] in the ALS model.

Masitinib underwent a Phase III trial with 394 participants, which compared Masitinib in combination with riluzole versus a riluzole-placebo. This study had broad inclusion criteria (FVC > 60%, disease onset < 3 years) and it was successful on its primary (ALSFRS-R) and secondary endpoints (ALSAQ-40, FVC, time-to-event) [284]. AB Science submitted a marketing application that was based on interim results from this trial; however, this application was declined due to low data reliability and possible bias [285]. A confirmatory Phase III trial (495 participants) using an optimized examination protocol will be launched to further evaluate and support the data gained from the first trial (trial ID#: NCT03127267).

## 14. Clinical Trials in the Past

**Figure 3:**
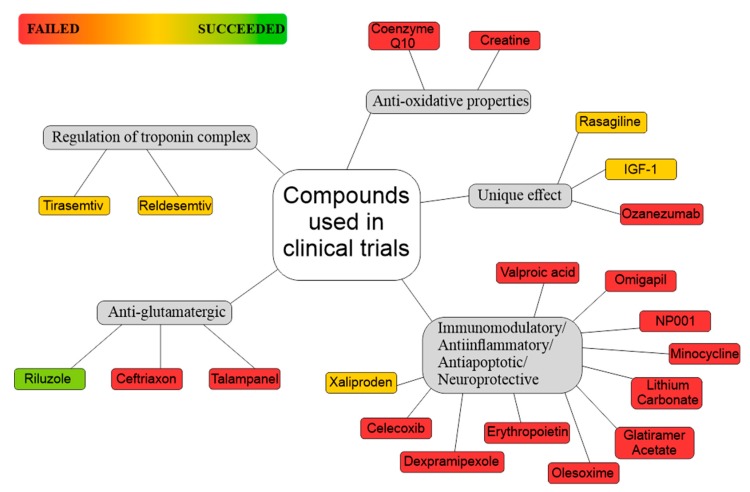
Compounds were divided into groups according to their effect, as described in clinical studies. In the category of anti-glutamatergic both Ceftriaxone [286] and Talampanel [287] failed. Another category of drugs are compounds that are thought to have anti-oxidative properties. Both Coenzyme Q10 [288,289] and Creatine [290,291,292,293,294] have failed to demonstrate clinical efficacy. Tirasemtiv and Reldesemtiv are able to regulate the release of calcium from the regulatory troponin complex and thus sensitize the muscle to calcium. Several trials assessed the efficacy of Tirasemtiv in human ALS patients. From all the studies [295,296,297,298], only one [298] managed to show statistical significance on two out of five secondary endpoints. Based on the results, a large-scale follow up study was launched but failed to demonstrate efficacy in all of its endpoints [299]. Efficacy of Reldesemtiv was assessed in a single Phase II trial. Although, this trial failed in its primary endpoint for all dose groups, patients showed a non-significant 27% reduction of decline in SVC. Moreover, all groups compared to the placebo, showed a significant 25% change in ALSFRS-R slope [300]. Most compounds used, are believed to have either immunomodulatory/anti-inflammatory/antiapoptotic and/or neuroprotective properties. Most of these compounds failed to demonstrate statistical significance in all of the studies´ endpoints. Celecoxib—[301], as part of a combined treatment with Creatine [302], Dexpramipexole [286,303], Erythropoietin [304,305], Glatiramer Acetate [306,307], Lithium Carbonate [308,309,310,311], Minocycline ([312,313,314] as part of a combined treatment with Creatine [302]), NP001 [315,316], Olesoxime [317], Omigapil (TCH-346) [318], and Valproic acid [319]. Xaliproden has managed to succeed in 1 out of 8 endpoints in one of two trials [320], however, both trials failed on their primary endpoints [320,321]. Trials of two compounds—Acthar gel (released in press in 2016) and Pioglitazone [322], were prematurely terminated due to potential risk to patients (Acthar gel) and futility (Pioglitazone). Other drugs are considered to have unique effects among the compounds assessed. Trials of Ozanezumab have failed to show clinical efficacy [323]. Another compound tested, insulin-like growth factor 1 (IGF-1), has failed to demonstrate clinical efficacy in three separate trials [324,325,326], even though one of the studies [325] succeeded in three out of three of its secondary endpoints. Another compound, Rasagiline, failed to show clinical efficacy in a single trial [327], however, post-hoc analysis has revealed a significant reduction of ALS progression in patients with an initial ALSFRS-R slope greater than one (patients with faster ALS progression).

The safety and efficacy of plenty of novel drugs have been evaluated over more than 25 years. Despite the fact that most of the tested drugs were proven to be clinically safe and tolerable by humans, the vast majority of the trials failed to demonstrate clinical efficacy of the tested compounds. In this review, we conducted a search for double-blind, randomized, placebo-controlled trials with 80 or more participants across the MEDLINE and clinicaltrials.gov databases. We then assigned every compound to a specific category, describing the effect of that compound. In Figure 3, there are compounds that already underwent a clinical trial, whereas, in Table 3, there are compounds that are currently tested or compounds used in clinical trials, which are recruiting participants.

## 15. Current Clinical Trials

Several compounds are currently being investigated as a potential treatment in large scale studies with ALS patients. Tauroursodeoxycholic acid (TUDCA) is an anti-apoptotic compound that competitively binds pro-apoptotic Bcl-2 proteins. TUDCA has been demonstrated to decrease apoptosis and increase survival in mouse models [328,329], and was also shown to have preliminary efficacy in a Phase II trial [330]. It is currently being evaluated in a Phase III trial (trial #ID: NCT03800524), as well as part of a combined treatment with phenylbutyrate (AMX0035; trial #ID: NCT03491462).

Two compounds with neuroprotective properties are also being tested. Fasudil was shown to be neuroprotective in one study in G93A mice [331], and there is currently an ongoing Phase II trial [332]. Pimozide is currently being evaluated in a Phase II trial (trial #ID: NCT03272503); however, a recent study in two different ALS mouse models has shown no efficacy on neuromuscular junction innervation or MN loss. On the contrary, it appears that Pimozide elevates the levels of misfolded SOD1 [333].

Methylcobalamin was shown to reduce the levels of homocysteine, which has been linked to neurotoxicity in ALS [334]. An early study [335] suggested an improvement in the motor functions while using ultra high dosage of methylcobalamin but a large-scale phase II/III study to assess its efficacy, launched in 2007 [336] was unsuccessful in fulfilling both primary and secondary endpoints. Later analyses have shown an improvement in patients, who received the treatment not later than 12 months following the diagnosis. Currently, a Phase III trial is recruiting new participants (NCT03548311).

Two compounds that are associated with the metabolism of specific metals are currently being tested. Deferiprone decreases levels of iron via chelation. A Phase II trial was conducted to assess safety and efficacy for the use in treating ALS. This study has shown positive results in terms of improvement in ALSFRS-R scale and body weight index [337]. Currently, the efficacy of deferiprone is evaluated in a Phase III study (240 participants, recruiting) with the primary endpoint being CAFS score that is based on changes in ALSFRS-R and survival (trial #ID: NCT03293069). The second compound, Cu(II)ATSM, is used to deliver copper to damaged cells. A Phase I trial (trial #ID: NCT02870634) succeeded in primary (safety) and secondary endpoints (ALSFRS-R, FVC, ECAS) [338]. Recent studies in both fALS and sALS mouse models have repeatedly shown neuroprotective and anti-inflammatory effects of Cu(II)ATSM [339,340]; a Phase II/III trial is currently enrolling participants (NCT04082832) and it will assess changes in ALSFRS-R scale, ECAS, and SVC scores.

The compound able to induce the production of heat shock protein 70 (HSP70)—Arimoclomol—is also undergoing clinical trials. The role of HSP70 as a chaperone is to reduce the levels of misfolded proteins, including mSOD1. A Phase II trial with 38 participants [341] has proved its safety and tolerability and non-significant efficacy in the secondary endpoints (ALSFRS-R, FEV6, CAFS). An additional Phase III study (231 participants—NCT03491462) was launched in 2018 (CAFS—survival, ALSFRS-R, SVC).

## 16. The Future of Clinical Trials

Ibudilast was primarily developed for bronchial asthma and post-stroke complications. However, its neuroprotective effect has recently been shown in a number of studies (reviewed by [342]). A Phase II ALS study (trial #ID: NCT02238626) reported success in achieving its primary endpoint (safety and tolerability) and secondary endpoints (ALSFRS-R, ALSAQ-5) [343]. Another Phase I/II study (trial #ID: NCT02714036) was carried out and completed earlier this year, but the results have not yet been published. A Phase II/III trial (230 patients, trial #ID: NCT04057898) assessing the efficacy of Ibudilast in ALS patients is currently recruiting participants.

The RNS60 was shown to have potential anti-inflammatory and neuroprotective effects in animal models of neurodegenerative diseases [344] and different Phase I trials have demonstrated RNS60 safety and tolerability. A single Phase I trial in ALS patients [345] has met its primary endpoints of safety and tolerability; however, no changes in the secondary endpoints (SVC, ALSFRS-R) were observed. A Phase II trial is currently recruiting new participants. The assessment of ALS biomarkers is its primary endpoint (trial #ID: NCT03456882).

### 16.1. Gene Therapies

Over recent years, a few approaches that are based on gene therapy have emerged for the treatment of ALS. The most promising and the most in-depth examined compound is BIIB067 (Tofersen), which was developed by Biogen. Tofersen is an antisense gene therapy. A piece of single strand DNA complementary to mSOD1 mRNA binds to it. The double strand DNA is then targeted for degradation, which leads to the reduction of mSOD1 levels. Preliminary data from Phase I/II study showed a statistically significant decrease in the levels of mSOD1 protein in CSF. The treated patients also showed a slow decrease in ALSFRS-R values, respiratory function, and muscle strength [347]. Recently (May 2019), Biogen launched a Phase III trial, which is recruiting new participants (trial #ID: NCT02623699).

Another gene therapy, miQure, targets mRNA of a different gene, C9orf72, with a set of two microRNAs. Again, this results in C9orf72 downregulation. miQure was shown to reduce the levels of faulty C9orf72 protein in cellular [348] and mouse models [349].

Gene therapy using VM202 was recently evaluated in a Phase I/II ALS trial. VM202 is a plasmid, which contains a sequence of two different hepatocyte growth factor (HGF) isoforms. HGF is a neurotrophic factor, which promotes neuronal growth and repair. The results from the trial show that VM202 is safe to use in ALS patients and the study also reported a decrease in ALSFRS-R decline [350]. A different compound currently in development, SynCav1, was shown to improve motor function and survival in a preclinical ALS mouse model [351].

### 16.2. Cell-Based Therapies

*Astrocyte-based therapies*. In recently published work, Izrael et al. described an original and promising therapeutic approach while using human embryonic stem cell (hESC)-derived astrocytes (hES-AS) [352]. Their findings suggested that intrathecal delivery of young and healthy astrocytes helps to overcome the neurotoxic function of endogenous astrocytes in ALS. In addition, the functional and secretome analyses confirmed that these astrocytes secrete soluble factors, which promote the growth of axons and neuron survival, and they are able to uptake extracellular glutamate and protect motor neurons from oxidative stress damage. A similar approach was described in a previous report showing the transplantation of astrocyte precursors leading to delayed progression of mutant SOD1-mediated disease in rodents [108]. On 24 September, 2019Kadimastem Ltd. (https://www.kadimastem.com) announced promising interim results of Phase 1/2a clinical trial (trial #ID: NCT03482050) for the treatment of patients with ALS. The primary objective of the trial was to evaluate the safety of injecting AstroRx^®^ cells(100 × 106 cells), containing functional healthy astrocytes that are derived from human embryonic stem cells (hESC), into the spinal cord fluid of ALS patients with the goal of supporting the malfunctioning cells in the brain and spinal cord. A secondary objective of the trial included preliminary efficacy. Of note, the interim results showed significant decline in disease progression, and no treatment-related serious adverse events or dose-limiting toxicities were reported. AstroRx^®^ has been granted orphan drug designation by the FDA. The final results of cohort A are expected by year-end 2019; the results of cohort B (five patients receiving higher dose of cells) are expected at the end of 2020. To the best of our knowledge, this is the only company developing astrocyte-based cell therapy for the treatment of ALS, which might become a breakthrough in ALS treatment.

In 2020, a Phase 1/2a open-label study (trail # ID: NCT02478450) will be initiated to investigate the safety of the transplantation of human glial restricted progenitor cells (hGRPs; Q-Cells^®^) into the lumbar/cervical spinal cord of ALS patients. These cells represent one of the earliest precursors within the oligodendrocytic- and astrocytic cell lineage and their usage in ALS therapy appears to be a beneficial approach resulting in the enrichment of nervous system by healthy donor-derived astrocytes. This study aims to obtain preliminary data on the safety, tolerability, and early efficacy of Q-Cells^®^ transplantation in subjects with ALS. Subsequent cohorts will receive escalating doses transplanted unilaterally in cervical spinal cord following an initial cohort receiving cell transplants unilaterally in the lumbar spinal cord. Subjects and outcome measure assessors will be blinded to the side of treatment. The application of lineage-restricted precursors in spinal cord injury already indicated that differentiation of GRPs into astrocytes before transplantation might be more advantageous in the context of axon regeneration.

The aim of another glia-based clinical trial (trial # ID: NCT02943850) is the safety of transplanting neural progenitor cells that have been engineered to produce a glial cell line-derived neurotrophic factor (GDNF) into the spinal cord of ALS patients to promote the survival of of neuronal cells. Here, they have been derived to specifically become astrocytes, and they are termed CNS10-NPC-GDNF. This study will be the first to use a genetically modified progenitor cells to treat a neurodegenerative disease. This is a Phase 1/2a, single-center, blinded, safety study of two escalating doses of human neural progenitor cells expressing GDNFdelivered unilaterally to the lumbar region in ALS subjects with moderate leg involvement.

*Targeting immune system*. Among the key players in the pathogenesis of ALS, microglia and T regulatory lymphocytes (Treg) are candidate cells for influencing onset/progression of the disease. Such therapeutic strategies targeting the modulation of key immune cells might result in switching the patient’s environment from a pro-inflammatory toxic to an anti-inflammatory, and neuroprotective condition. There are several clinical trials using autologous infusion of expanded Treg cells (also combined with IL-2 injections) during early and late phases of ALS disease in patients with varying rates of disease progression (trial #ID: NCT03241784, NCT04055623). Similarly, single cycle of repeated intrathecal injections of bone-marrow MSCs also demonstrated a clinical benefit lasting at least six months, as they regulate innate and adaptive immune cells, through the release of soluble factors such as TGF-β and elevation of regulatory T cells (Tregs) and T helper-2 cells (Th2 cells). Cytokine profiles of cerebrospinal fluid provided evidence that MSCs have a role in switching from pro-inflammatory to anti-inflammatory conditions.

A further interesting therapeutic approach is based on data that are obtained in mouse models, which have shown an altered enteric flora in early ALS stages and, thus, suggesting the role of gut microbiota (GM) in ALS pathogenesis. The GM mainly acts on shaping immune tolerance and regulating the number of Treg cells. Fecal microbial transplantation (FMT) is a well-known therapeutic intervention used to re-establish the proper microenvironment and modulate enteric and systemic immunity. The aim of this clinical trial is to perform a multicenter randomized double-blind study employing FMT as a therapeutic intervention for ALS patients (NCT0376632). The expected outcome is an increase in Treg cell number and a switch of the immune system that surrounds MNs to an anti-inflammatory, neuroprotective status. Simultaneously, extensive analyses on immune cell populations, cytokines levels, and microbiota should elucidate early processes, which possibly lead to the degenerative ALS. Validated clinical outcomes of ALS (survival, forced vital capacity, and modifications in ALSFRS-R), besides safety and quality of life, are included as the secondary aims of the trial. Of note, this is the first trial with FMT as a potential intervention to modify immunological response to ALS and disease progression at an early stage [353]. 

*Stem-cells-based therapy.* Trials with different kinds of stem cells have been conducted over the past years. The majority of these studies provided mixed results due to being underpowered (for an excellent review see [354]). One of the treatments, NurOwn, is evaluated in a large-scale trial. This is a therapy using bone marrow mesenchymal stem cells. Initially, two small scale studies (Phase I/II and Phase IIa) showed that the treatment with NurOwn is safe and well-tolerated with only mild and temporary side effects. Both of the studies also showed a tendency in ALSFRS-R ad FVC score improvement [355]. Later, a larger Phase II trial (48 participants) produced similar results and showed an improvement in the ALSFRS-R and FVC scores, with no adverse events. A reduction of inflammatory activity was also observed [356]. A Phase III trial (200 participants) finished recruiting all of the participants in October 2019, with ALSFRS-R as a primary endpoint and measuring the levels of biomarkers in serum and CSF as a secondary endpoint. The current results show that NurOwn might be more beneficial for patients with rapid ALS progression. There has also been a report of an increased level of specific miRNAs in the CNS, which are associated with increased immunomodulatory and neuroprotective activity [357]. An additional clinical trial was focused on evaluating the safety, tolerability and therapeutic effects of transplantation of escalating doses of autologous cultured mesenchymal bone marrow stromal cells secreting neurotrophic factors (MSC-NTF), in ALS patients (trial# ID: NCT01777646) comprising multiple intramuscular injections at 24 separate sites, in addition to a single intrathecal injection into the cerebrospinal fluid.

## 17. Conclusions

In conclusion, the current level of knowledge of ALS pathology shows a distinctive turn of scientific approach during the past years. A previous focus on MNs and symptomatic treatment was replaced by an attempt for deeper understanding of the molecular processes within the brain tissue and spinal cord. Such a turn brought scientists to glial cells and their strong impact on the functioning of MNs during ALS progression. It was discovered that all types of glial cells are affected by ALS pathology and they themselves can be the reason for the fast progression of ALS. As previously mentioned, glial cells have a substantial effect on MN functioning and survival, as they are able to damage MNs by the non-cell autonomous effect.

Based on the results of basic research, numerous molecules were tested in clinical trials that had an impact on glial cells and had the potential to improve patients’ survival. The pathway to the development of reliable treatment was long and comprised of many failures. However, recently, some clinical trials that focused on the properties of glial cells were successful. Riluzole and Edaravone are substances that are currently used by clinicians. Unfortunately, they only provide a mild improvement in terms of patients’ survival. However, there are other compounds being tested and there is the possibility of their approval for the treatment of ALS. One of them is Masitinib, which already demonstrated clinical efficacy and it is likely to be approved for clinical usage in the near future. Based on our findings from literature, we are optimistic regarding the potential of other glia-directed compounds, such as Cu(II)ATSM and Ibudilast, which have succeeded in primary studies and they are currently being evaluated in large-scale trials. We also recognize great potential in the new state-of-the-art methods, such as gene therapy and approaches that are based on stem cells. These could be beneficial, especially for the treatment of some forms of fALS.

## Figures and Tables

**Table 1:** Summary of representative SOD1 animal models of ALS.

Species	Protein/Gene	Protein Function	Mutation	Promoter	CNS Overexpression (Fold)	Symptoms Onset (Weeks)	Survival (Weeks)	Phenotype	Ref.
Mouse	Cu-Zn SOD/*Sod1*	ROS detoxification	D90A	human SOD1	20	52	61	ALS-like phenotype, progressive MN deficit, axonal degeneration, paralysis, gliosis, SOD1 inclusions, mitochondrial vacuolation	
			G37R	human SOD1	4–12	15–17	25–29		[72,73]
			G85R	human SOD1	0.2–1	35–43	37–45		[74]
			G86R	human SOD1	n/a	13–17	17		[75]
			G93A	human SOD1	8	12	40–50		[76]
			G127X	human SOD1	0.5–1	35	36		[77]
			H46	human SOD1	n/a	20	24		[78]
			H46R/H48Q	human SOD1	n/a	17–26	n/a		[79]
			T116X	human SOD1	n/a	41	43		[80]
			L126X	human SOD1	0–0.5	28–36	n/a		[81]
			L126delTT	human SOD1	2	17	18		[82]
Rat	Cu-Zn SOD/*Sod1*	ROS detoxification	H46R	human SOD1	6	20	24	ALS-like	[83]
			G93A	human SOD1					
Zebrafish	Cu-Zn SOD/*Sod1*	ROS detoxification	G93R	zebrafish SOD1	3	48	72–108	Muscle athrophy, MN loss, œsurvival	[84]
Drosophila	Cu-Zn SOD/*Sod1*	ROS detoxification	A4V		3–5	4	normal	glia activation, no MN loss	[85]
			G85R		1–2	2	normal		
C. elegans	Cu-Zn SOD/*Sod1*	ROS detoxification	G85R			4 days	decreased	SOD1 aggregates, no MN loss	[86]
			H46R/H48Q			4 days			
Dog.	Cu-Zn SOD/*Sod1*	ROS detoxification	E40K/E40K	endogenous	1	5 years	6–9 months from symptom onset	DM, axonal lesions, functional deficit in UMN, LMN	[87]

ALS—amyotrophic lateral sclerosis; ROS—reactive oxygen species; SOD—superoxid dismutase; UMN—uper motor neurons; LMN—lower motor neurons; DM—degenerative myelopathy.

**Table 2:** Summary of representative TDP-43 animal models of amyotrophic lateral sclerosis (ALS).

Species	Protein/Gene	Protein Function	Mutation	Promoter	CNS Overexpression (Fold)	Symptoms Onset (Weeks)	Survival (Weeks)	ph-TDP-43	Inclusions	Phenotype	Ref
Ubiquitous expression											
Mouse	TDP-43/*TARDBP*	RNA metabolism	A315T	mPrP	3	13	22		TDP-43+ Ub+	MN degeneration, gliosis	[88]
			A315T	mPrP	4	4	37.5	+	TDP-43+ Ub+	gliosis, muscle atrophy	[89]
			A315T	human TDP43 (BAC)	3	7 months	>12 months		TDP-43+ Ub+	motor and cognitive phenotype, NMJ denervation, gliosis	[90]
			G348C	human TDP43 (BAC)	3	7 months	>12 months		TDP-43+ Ub+		
			M337V	mPrP	2.5	3	10	+	TDP-43+ Ub+	NII/NCI, motor dysfunction, gliosis, mitochondria aggregation	[91]
			M337V	mPrP	1–1.5	10 months	>17 months	-	-	motor dysfunction, ↓ NMJ integrity, axonopathy, gliosis	[92]
			Q331K	mPrP	1–1.5	3 months	>17 months	-	-		
Rat	TDP-43/*TARDBP*	RNA metabolism	M337V	human TDP43 (BAC)	high	3	4–8			MN degeneration, muscle atrophy	[57]
Zebrafish	TDP-43/*TARDBP*	RNA metabolism	A315T							motor defects, axonopathy, aberrant branching	[60]
Drosophila	TDP-43/*TARDBP*	RNA metabolism	Q331K				lethal			motor deffects	[93]
			M337V				lethal				
			G348C							abberant branching	[94]
			A315T								
C. elegans	TDP-43/*TARDBP*	RNA metabolism	M337V							locomotor deffects, paralysis, MN degeneration	[95]
			A315T								
Neuron specific expression											
Mouse	TDP-43/*TARDBP*	RNA metabolism	ΔNLS	CaMK2	7.9	1		+		motor and cognitive phenotype, gliosis	[96]
			M337V	Thy1.2	1.7	11 days	3	+	TDP-43+ Ub+	motor defects, MN degeneration, gliosis	[97]
Rat	TDP-43/*TARDBP*	RNA metabolism	iTDP43-M337V	Chat NEFH		2	60 days		TDP-43+ Ub+	MN degeneration, muscle atrophy, gliosis	[98]

TDP-43—TAR DNA-binding protein 43; mPrP—murine prion protein; ph-TDP-43—phospho-TDP-43; CaMK2—Ca^2+^/calmodulin-dependent protein kinase II; Chat—choline acetyltransferase; NEFH—neurofilament heavy polypeptide; NMJ—neuromuscular junction.

**Table 3:** Currently tested compounds and compounds used in studies that are recruiting participants.

Compound	Endpoints ^§^	Outcome of Previous Studies in ALS Patients	Reference
TUDCA	ALSFRS-R	success	[330]
Fasudil	survival, ALSFRS-R	-	[332]
Pimozide	ALSFRS-R, SVC	failure	[346]
Methylcobalamin	survival, ALSFRS-R	success	[336]
Deferiprone	CAFS	Mixed *	[337]
Cu(II)ATSM	ALSFRS-R, ECAS, SVC	success	-
Arimoclomol	CAFS	Failure **	[341]
Ibudilast	ALSFRS-R, ALSAQ-5	success	-
RNS60	SVC, ALSFRS-R	failure	[345]

§—primary endpoints, secondary endpoints in studies which primarily assessed safety and tolerability. *—study reported statistically significant differences in ALSFRS-R and body-mass index (BMI) decline between the three-month treatment-free period compared to the first three months of treatment. **—this study has shown a favorable trend in decreasing the decline of ALSFRS-R, FEV6 (Forced Expiratory Volume in six seconds), and CAFS, although this trend was not statistically significant.
